# Visualization of stress fractures of the foot using PET-MRI: a feasibility study

**DOI:** 10.1186/s40001-015-0193-6

**Published:** 2015-12-23

**Authors:** Moritz Crönlein, Isabel Rauscher, Ambros J. Beer, Markus Schwaiger, Christoph Schäffeler, Marc Beirer, Stephan Huber, Gunther H. Sandmann, Peter Biberthaler, Matthias Eiber, Chlodwig Kirchhoff

**Affiliations:** Department of Trauma Surgery, Klinikum rechts der Isar, Technical University of Munich, Ismaninger Strasse 22, 81675 Munich, Germany; Department of Nuclear Medicine, Klinikum rechts der Isar, Technical University of Munich, Ismaninger Strasse 22, 81675 Munich, Germany; Musculoskeletal Imaging Department of Radiology, Kantonsspital Graubünden, Loëstrasse 170, 7000 Chur, Switzerland; Department of Radiology, Klinikum rechts der Isar, Technische Universität München, Ismaninger Str. 22, 81675 Munich, Germany; Clinic for Trauma and Reconstructive Surgery, BGU Tübingen, Schnarrenbergstraße 95, 72076 Tübingen, Germany; Department of Nuclear Medicine, University Hospital Ulm, Albert-Einstein-Allee 23, 89081 Ulm, Germany

**Keywords:** Stress fractures, Stress reactions, PET-MRI, Foot pain, Microfractures

## Abstract

**Background:**

Diagnosis and treatment of stress fractures still remains to be a clinical and radiological challenge. Therapeutic options vary from conservative treatment to surgical treatment without a clear treatment concept. Recently the combination of PET and MRI has been introduced, aiming a superior diagnostic accuracy in clinical practice. Therefore the aim of our study was to analyse whether PET-MRI would be a feasible technique to recognize stress fractures of the foot and to analyse if our conservative treatment plan leads to a good clinical outcome.

**Methods:**

Therefore, 20 patients with suspected stress fractures of the foot and ankle underwent plain radiography and ^18^F-Fluoride PET-MRI. Two blinded readers assessed in consensus both imaging techniques for the presence of stress fracture, stress reaction or osteoarthritis. Patients with stress fractures or stress reactions in the foot and ankle area underwent our conservative treatment plan, with immobilization in a VACO®ped cast for 6 weeks under partial weight bearing on forearm crutches. The benefit of our conservative therapeutic concept was evaluated by the patients on the basis of VAS and FAOS scoring systems before and after treatment.

**Results:**

8 out of 20 patients underwent conservative treatment after diagnosis of either a stress fracture or a stress reaction of the foot and ankle area. PET-MRI identified four stress fractures and seven stress reactions. In all cases, no pathological findings were present on plain X-ray. FAOS and VAS significantly improved according to the patients’ records.

**Conclusions:**

PET-MRI seems to be a useful modality to diagnose stress fractures and stress reactions of the foot and ankle area, especially when conventional modalities, such as plain radiographs fail. Conservative management is a promising therapeutic option for the treatment of stress fractures. To rule out the benefits compared to a surgical treatment plan, further studies are needed.

## Background

Due to the increasing number of recreational athletes, sports-related injuries represent a significant challenge in orthopaedic medicine [1, 2]. Among these injuries stress-related fractures (SFX) have gained increased attention in the recent past. Every loading exposes bone to internal forces of stress and deformation or strain. SFX occur due to repetitive overuse and/or overload, when stress-induced microfractures exceed the remodelling capacity of the bone and accumulate into macrofractures [3, 4]. Further factors contributing to SFX involve hormonal, metabolic and nutrition components [5]. Also anatomical predilections like leg length differences, pes planus/cavus and high Q-angle may increase the risk. SFX have been described in different parts of the human skeleton but are most common in the lower extremity areas of the tibia (23.6 %), navicular (17.6 %), metatarsals (16.2 %), femur (6.6 %) but are also reported in the fibula, navicular, sesamoid, sacrum (1.6 %) and ribs [4, 6]. Therefore SFX of the lower extremity account for 80–90 % of all SFX, representing between 0.7 and 20 % of all sports-related injuries. They are commonly observed among individuals who participate in endurance, high load-bearing activities with the highest incidences of up to 15 % observed in long-distance and track athletes followed by gymnasts and field athletes [5].

In orthopaedic sports medicine, the fracture site is crucial for the therapeutic management. Stress injuries are classified as either high-risk or low-risk injuries according to their location and the associated anatomic preconditions [5, 7]. Depending on the grading, the therapeutic options in case of SFX vary. Conservative treatment involves restriction of weight bearing, physical activity and, depending on severity, lasts from 6 weeks to over 6 months [3]. Operative treatment is only described as second choice in current literature [8, 9].

Besides the requirement of long periods of recovery, the functional outcome is often complicated by delays in diagnosis. Accurate diagnosis of SFX is based on the patient’s history, the anatomical area and different imaging techniques [10]. Regardless, early recognition is the optimal goal to minimize the potential for microfractures to turn into macrofractures. Standalone X-ray provides a sensitivity ranging from 12 to 56 %, while specificity ranges from 88 to 96 %, implying that many stress fracture diagnoses might be missed [3]. In contrast magnetic resonance imaging (MRI), the current gold standard in literature reveals a sensitivity of 68–99 %, while specificity ranges from 4 to 97 % [3, 6, 10]. Summarizing MRI currently is the most effective imaging modality for diagnosing SFX. However, it carries the potential of overdiagnosis and treatment, i.e. longer restriction of weight bearing in simple bone oedema or osteoarthritic degeneration.

Recently, positron emission tomography (PET) MRI, as a combination of morphological and functional imaging, has been introduced in clinical practice [9, 11, 12]. This new technique offers, besides the information on soft tissue and bone marrow pathology, an additional diagnostically relevant information on the bone metabolism when using 18F-Fluoride as a radiotracer.

Therefore, the aim of our study was to analyse, whether ^18^F-Fluoride PET-MRI would be a feasible technique to recognize stress fractures of the foot and to analyse if a conservative treatment plan leads to a good clinical outcome in patients suffering from foot and ankle pain due to acute fractures or stress reactions.

## Methods

### Patients

Between February 2012 and March 2013, 20 patients with suspected stress fractures of the ankle/foot were prospectively enrolled. Inclusion criteria were: localized pain in the foot and ankle area for at least 6 weeks without an adequate trauma in the patients’ history, X-rays of the affected area without verification of a fracture line and written informed consent to undergo ^18^F-Fluoride PET-MRI examination. The study was approved by the local institutional review board of Klinikum rechts der Isar (reference number: 2967/10). Exclusion criteria were: age under 18, pregnancy and contraindications for ^18^F-Fluoride PET-MR imaging. A comparison between ^18^F-Fluoride PET/MR and ^18^F-Fluoride PET/CT in these patients has been recently published [13]. However, this previously published study only showed theoretical aspects of the use of ^18^F-Fluoride PET/MR for the diagnosis of unclear foot pain, the present study examines the practical use of ^18^F-Fluoride PET/MR for the diagnosis of stress fractures, as a delayed diagnosis can lead to multiple complications like prolonged pain or non-unions [14].

### Diagnostic and therapeutic regime

In case the patients matched the inclusion criteria and approved their written consent, the Foot and Ankle Outcome Score (FAOS) and the Visual Analogue Scale (VAS) were determined. The patients were then submitted to the department of nuclear medicine, where ^18^F-Fluoride PET-MR imaging was performed. PET-MR images were analysed in consensus by a dual-board-certified radiologist and nuclear physician with several years of experience in PET-imaging reading and a board-certified radiologist with special training in musculoskeletal radiology. The criteria for the diagnosis of osteoarthritis, stress reaction and stress fracture using ^18^F-Fluoride PET-MR were recently published [13].

After the analysis of the PET-MR scans, the patients were examined in the outpatient clinic, where the results were discussed. Only the patients who showed either stress fractures or stress reactions in the PET-MR scans underwent our conservative treatment plan, all other patients were not included in our follow-up examinations. Each patient with stress fractures/stress reactions in the ^18^F-Fluoride PET-MRI-Scan received a VACOped® cast and was mobilized with partial weight bearing (15–20 kg) with forearm crutches for 6 weeks. After 12 weeks, the patients were examined in the outpatient clinic again, the FAOS and VAS were determined for a second time.

### Questionnaire

The Foot and Ankle Outcome Score (FAOS) consists of 42 Likert Scale questions. It is divided into five separate subscales: symptoms (7 questions), pain (9 questions), function (17 questions), sports performance (5 questions) and quality of life (4 questions). Results range from 0 to 100 points. A score of 0 indicates poor points, a score of 100 indicates the best score [15, 16].

### ^18^F-Fluoride PET-MRI

All of the ^18^F-Fluoride PET-MR examinations were conducted on a whole-body hybrid ^18^F-Fluoride PET-MR system (Biograph mMR; Siemens Healthcare, Erlangen, Germany). For attenuation correction, a coronal 2-point Dixon 3D volumetric interpolated examination (VIBE) T1-weighted (T1w) MR sequence was acquired. Together with the start of this Dixon MR sequence, the PET acquisition (20 min) started simultaneously in the same BP, thus ensuring optimal temporal and regional correspondence between MRI and PET data. Additionally, a dedicated MR protocol of the foot was defined depending on the localization of the maximum pain with the following parameters: slice thickness 3 mm, field of view (FoV) 120–225 mm, matrix: 320 × 256–384 × 384. The protocol consisted of at least one intermediate-weighted fat-saturated (PDfs) sequence in two planes and one T1- and T2-weighted turbo spin echo (TSE) sequence.

### Statistical analysis

Data are given in mean values (arithmetic mean) and standard deviations. For the comparison of the FAOS score, the paired *t* test was performed with the software SigmaStat Version 3.5 (Systat Software, Inc., San Jose, California, USA).

## Results

Between February 2012 and March 2013, 20 patients were included in our study. Eight patients of our study group were identified with either stress fractures or stress reactions by ^18^F-Fluoride PET-MRI. 7 of these 8 patients showed stress reactions and stress fractures at the same time, 1 patient showed an isolated stress fracture without a concomitant stress reaction. The priorly performed X-rays of these patients showed no pathological findings (see Table 1).

**Table 1:** Overview about the radiological findings and the outcome after treatment based on VAS and FAOS scoring scales

Patient number	Sex	Age (years)	X-ray findings	^18^F-Fluoride PET-MRI findings	FAOS (before treatment)	VAS (before treatment)	FAOS (after treatment)	VAS (after treatment)
1	Female	20	No specific findings	Soft tissue oedema				
2^a^	Male	25	No specific findings	Stress fracture base OMT V	41.7	3	50	1
3^a,b^	Female	49	No specific findings	OMT I: stress fracture, OMT IV: stress reaction	24.75	4	45	2
4	Male	29	No specific findings	Tenosynovitis of extensor compartment				
5^b^	Female	45	No specific findings	Caput OMT III/IV: stress reaction	20	3	35	1
6	Female	60	No specific findings	Caput tali: stress reaction				
7	Male	28	No specific findings	Unremarkable				
8	Female	78	Osteoarthrosis of the ankle joint	Osteoarthrosis of several joints				
9^b^	Female	64	No specific findings	Anterior calcaneal process: stress reaction	23.75	3	29.75	3
10^b^	Male	61	No specific findings	Caput OMT II: stress reaction, osteoarthrosis of multiple joints	30.75	4	38	2
11	Female	68	Osteoarthrosis of the ankle joint	Osteoarthrosis of several joints				
12	Female	54	No specific findings	Osteoarthrosis after trimalleolar fracture				
13^a,b^	Female	58	No specific findings	Cuboid: acute stress fracture; anterior calcaneal process: stress reaction; calcaneus: old stress fracture	48.75	3	64.25	1
14	Male	72	No specific findings	Osteoarthrosis of several joints				
15^a,b^	Female	26	No specific findings	OMT III: stress fracture, sesamoid bone: stress reaction	45.7	5	45.7	2
16	Male	22	Bone cyst	Aneurysmatic bone cyst				
17	Male	27	No specific findings	Talus: osteochondral lesions				
18	Male	55	Osteoarthrosis of the ankle joint	Osteoarthrosis of multiple joints				
19	Male	74	Osteoarthrosis of the ankle joint	Osteoarthrosis of the ankle joint				
20^b^	Female	67	No specific findings	Anterior calcaneal process: stress reaction	52	4	62	1

The 20 patients that have been included in our study are listed with age and gender. Primary radiological findings (X-ray and PET-MRI) are listed as well as the FAOS and VAS scores before and after our conservative treatment

^a^Stress fracture

^b^Stress reaction

The other patients (*n* = 12) had no specific findings besides osteoarthrosis, neither in the PET-MRI nor in the other modalities. Eight patients of our study population underwent conservative treatment as described above. FAOS significantly improved from 35.9 ± 12.6 before treatment to 51.7 ± 12.5 after treatment (*p* ≤ 0.001). Pain also significantly improved from 3.6 ± 0.7 before treatment to 1.6 ± 0.74 after treatment (*p* ≤ 0.001) measured by to the Visual Analogue Scale (VAS) (see Table 1).

**Fig. 1:**
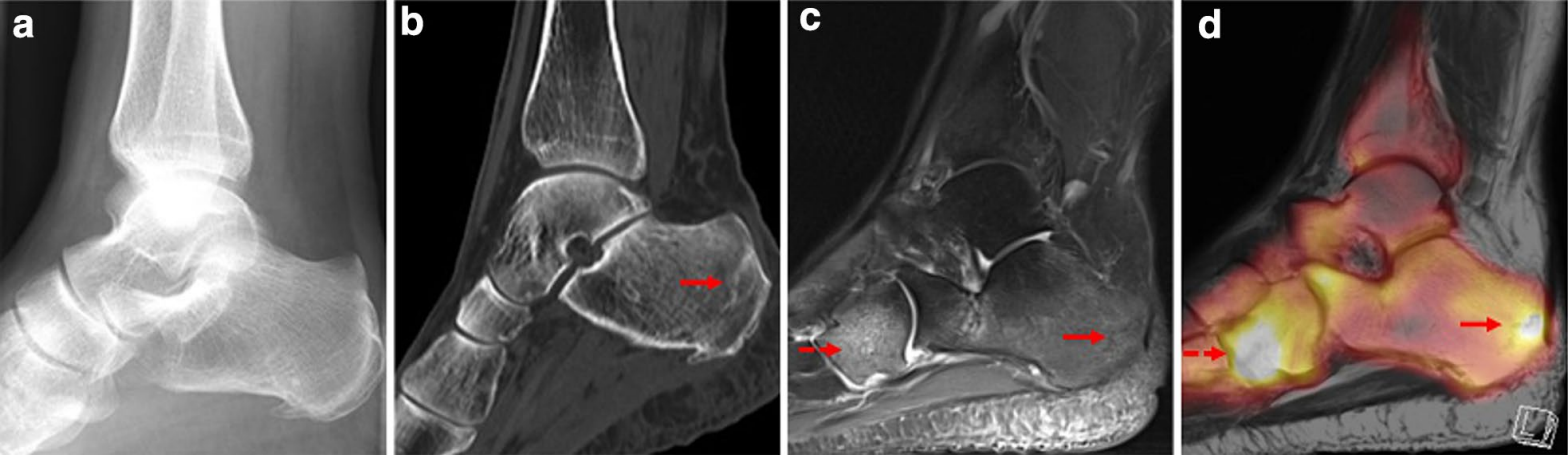
X-ray (**a**), CT scan (**b**), MRI scan (**c**) and ^18^F-Fluoride PET-MR (**d**) images of a 58-year-old female patient (patient no. 13). In the lateral X-ray (**a**) of the ankle region, neither acute stress fractures, nor degenerative lesions can be diagnosed. Sagittal CT images (**b**) show sclerotic lesions in the dorsal calcaneus (*red arrow*) and degenerative changes in the talonavicular region, no acute stress fractures are shown. Sagittal MRI images (**c**) show a fracture line in the dorsal calcaneus with little oedema (*red arrow*), due to an older stress fracture, coming along with a higher fluoride uptake in the ^18^F-Fluoride PET-MR (**d**, *red arrow*). Another stress fracture is shown in the mediodorsal parts of the cuboid with bone marrow oedema in the MRI scan (**c**, *dotted arrow*) and a higher fluoride uptake in the ^18^F-Fluoride PET-MR (**d**, *dotted arrow*)

**Fig. 2:**
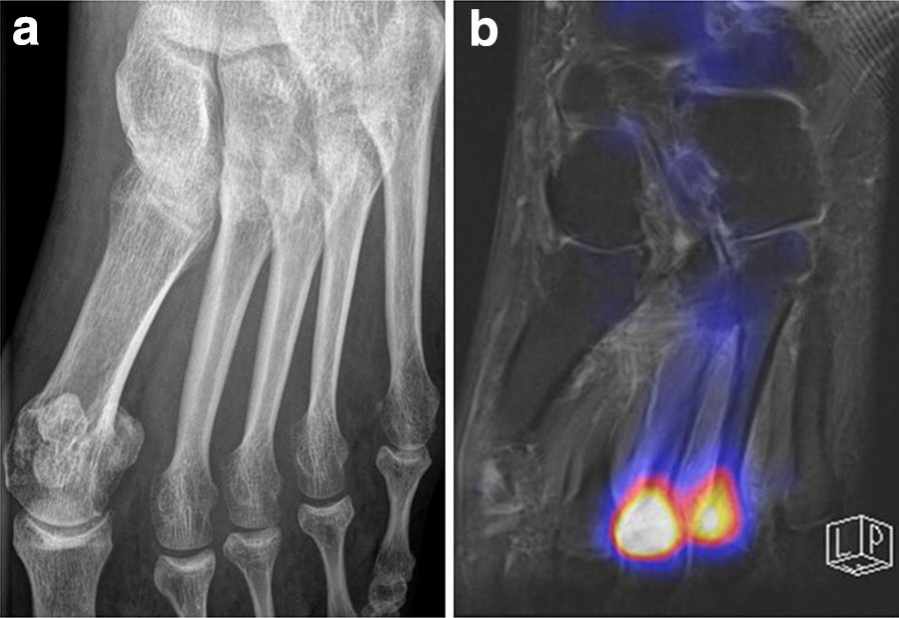
X-ray (**a**) and ^18^F-Fluoride PET-MR (**b**) images of a 45-year-old female patient (patient no. 5). The a.p. X-ray (**a**) of the left foot shows no acute stress fractures, degenerative changes in the tarso-metatarsal and metatarsophalangeal joints are illustrated. ^18^F-Fluoride PET-MR (**b**) show high tracer uptake in OMT III/IV heads suitable to an acute stress reaction, no fracture lines can be detected

Diagnostic imaging of two patients who underwent conservative treatment is shown exemplarily in Figs. 1, 2. Figure 1 illustrates the X-ray (a), CT (b), MRI (c) and ^18^F-Fluoride PET-MRI (d) scans of a 58-year-old female patient. She complained about progressive pain in the calcaneocuboid joint and the dorsal calcaneus for several months, without an adequate trauma in her history. The pain had an intensity of 3 on the Visual Analogue Scale (VAS), the FAOS was 48.75 before treatment. No relevant medical findings were seen on the X-ray, the sagittal CT images (b) showed sclerotic lesions in the dorsal calcaneus and degenerative changes in the talonavicular region. With the help of ^18^F-Fluoride PET-MRI a stress fracture in the dorsal calcaneus and the mediodorsal parts of the cuboid, with little bone marrow oedema (c), coming along with a higher fluoride uptake in the ^18^F-Fluoride PET-MR (d) could be detected. After conservative treatment, the patients’ situation improved. Pain was reduced by two points (3 to 1) on the VAS and the FAOS increased up to 64.25 points. Figure 2 shows the X-ray and PET-MRI scans of a 45-year-old female patient with progressive pain in the third and fourth metatarsal bone (OMT) after operative treatment of a Weber B fracture of the same ankle. 10–12 weeks after the operation, when the patient started to be more active, the pain occurred without any signs of trauma. A VAS of 3 and a FAOS of 23.75 were described before treatment (see Table 1). The X-ray showed slight degenerative changes, whereas acute stress reactions in the OMT III and IV heads were detected by ^18^F-Fluoride PET-MR with high tracer uptake (see Fig. 2). With our conservative treatment, a reduction of pain (VAS 3 to VAS 1) and an increased FAOS (up to 35 points) could be achieved.

## Discussion

Our study showed that ^18^F-Fluoride PET-MR can be useful in the diagnostics of stress fractures. 8 out of 20 patients with unclear foot/ankle pain were either diagnosed with “stress fractures” or with “stress reactions”. All of these patients underwent conservative treatment, following which a significant improvement of the FAOS and a pain reduction in the VAS could be achieved.

With the help of ^18^F-Fluoride PET-MRI, eight patients with relevant bony lesions could be identified in our study. Plain radiography showed no suspicious lesions in these cases. However, ^18^F-Fluoride PET-MR clearly illustrated even the small lesions directly due to a higher tracer uptake (^18^F-Fluoride PET-MR) and indirectly due to the bone marrow oedema, symbolizing large impact on the bone (MRI).

Looking at the current literature, different modalities such as plain radiography, computed tomography, scintigraphy and MRI are frequently used to diagnose stress fractures [6, 17, 18]. Even with the help of more than one single modality, diagnosis still remains a challenge [3]. Radiographs of the foot and ankle are often used as first-line diagnostic feature [19, 20]. Although X-rays are easy and fast to perform with only little amounts of radiation for the patient, there are limits when it comes to the diagnosis of small bony lesions. Especially in the early stages, only one-third of the fractures show typical radiographic signs [20, 21]. Local sclerosis and dense lines can only be observed as indirect fracture signs a few weeks after trauma with plain radiographs [17]. Therefore, additional imaging can be helpful.

Another widely accepted diagnostic feature—especially in the initial diagnostic phase—is computed tomography. Shearman et al. reviewed eight cases of longitudinal tibial stress fractures, respectively with respect to the ideal diagnostic modality. All of the patients had received normal plain radiographs initially. Additional CT imaging was performed in three cases and showed characteristic cortical lesions [20]. A high specificity of CT imaging in the diagnosis of stress reactions is also described by Gaeta et al. [19]. However, regarding the sensitivity of the diagnosis, MRI had advantages to CT [19, 22]. Fredericson et al. performed radiographs, scintigraphy (technetium bone scan) and MRI scans in fourteen runners with symptomatic leg pain and revealed that the MRI findings correlated with the scintigraphy, but the exact anatomical region of the lesion could be defined more precisely by MRI than by scintigraphy [23]. These findings are also confirmed by Miller et al. [24]. Scintigraphy itself shows a high sensitivity besides a very low specificity in the diagnosis of stress fractures [25]. Diagnosis of stress reactions remains to be a very big challenge. Apart from the high diagnostic effort, ^18^F-Fluoride PET-MRI, as an additional diagnostic tool, seems to be a suitable modality combining the high sensitivity of ^18^F-Fluoride PET with the high specificity of MRI in patients with suspected stress fractures.

After diagnosis, our patients underwent conservative treatment. They were immobilized in a VACOped® Cast for 6 weeks under partial weight bearing with 15–20 kg on forearm crutches. Although multiple different therapeutic strategies exist in current literature [3, 9], the ideal treatment of stress fractures still remains to be a big problem nowadays. Depending on the specific sites of occurrence, stress fractures can be classified into low-risk and high-risk fractures [5]. While low-risk stress fractures have a good chance to heal with conservative treatment, high-risk fractures are prone to delayed unions or non-unions more often [7]. An operative regimen with open reduction and internal fixation (ORIF) has been described by a few authors in current literature [26, 27]. Rongstad et al. showed the benefits of operative treatment of fourth metatarsal stress fractures in a retrospective study on 14 patients. 11 of the 14 patients chose operative treatment (ORIF) and returned to sports at an average of 12 weeks post surgery and would choose surgery for this kind of fracture again [27]. Nevertheless, an operative regimen is discussed controversially in current literature [10, 28] and a conservative regimen is preferred in most of the cases [1, 18, 29]. Operative treatment after failed fracture healing is described by Karthik et al. in a case of bilateral scapular spine stress fractures, where one side had united with conservative treatment and the other side had to be operated because of pain and non-union. The patient was asymptomatic at the final follow-up on both sides 2 years posttraumatically [30]. The ideal therapy of stress fractures is not found yet. We have good experiences with our conservative treatment regimen. Conservative therapy as first-line therapy is supported by many authors especially in low-risk stress fractures [1, 5, 7]. Operation after failed conservative treatment seems to be a good therapeutical concept.

## Conclusions

Stress fractures pose a challenge for modern medicine. Modern imaging techniques such as ^18^F-Fluoride PET-MR seems to be useful for diagnosis, especially when conventional methods do not detect the reason for unclear foot pain. Depending on the fracture site, the functional claim and the age of the patient, the right treatment regimen should be determined. For evaluating the benefits of operative vs. non-operative treatment, further studies are needed.

## Abbreviations

CT: computed tomography
FAOS: foot and ankle outcome score
MRI: magnetic resonance imaging
OMT: metatarsal bone
ORIF: open reduction internal fixation
PET: positron emission tomography
SFX: stress-related fractures
VAS: visual analogue scale
